# Numerical Expedition on the Potential of AgBiS_2_-Based Thin Film Solar Cells Employing Different Carrier Transport Layers

**DOI:** 10.1021/acsomega.4c02375

**Published:** 2024-08-06

**Authors:** Sabbir Ahmed Sayeem, Mst. Aysha Siddika, Sangita Rani Basu, Bipanko Kumar Mondal, Jaker Hossain

**Affiliations:** † Department of Electrical & Electronic Engineering, Pundra University of Science & Technology, Bogura, Bogura 5800, Bangladesh; ‡ Department of Electrical and Electronic Engineering, University of Chittagong, Chattogram 4331, Bangladesh; § Department of Electrical and Electronic Engineering, Begum Rokeya University, Rangpur, Rangpur 5400, Bangladesh; ∥ Solar Energy Laboratory, Department of Electrical and Electronic Engineering, University of Rajshahi, Rajshahi 6205, Bangladesh

## Abstract

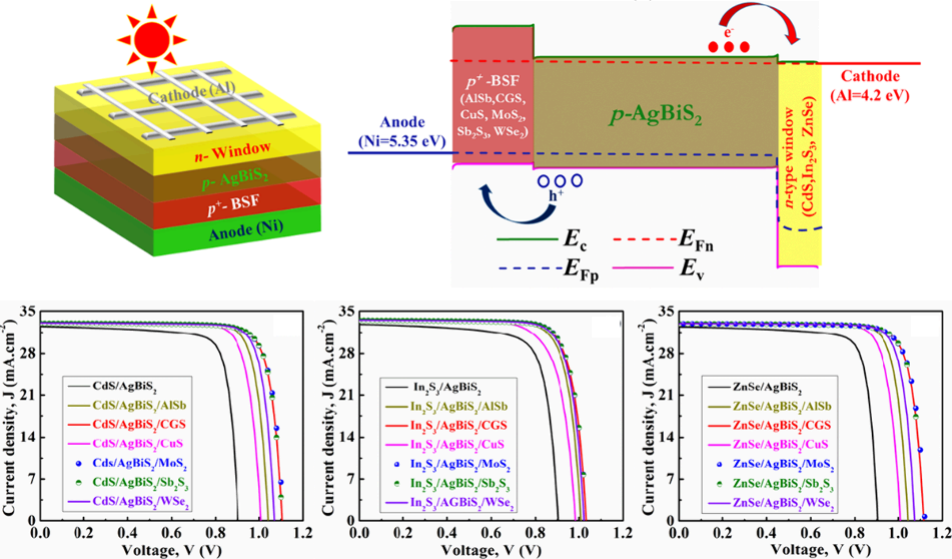

In this study, a photovoltaic (PV) device has been developed by using AgBiS_2_ as the key material. The simulation of the photovoltaic cell has been performed using the SCAPS-1D simulator to analyze the impact of each layer. The design incorporates three window layers, CdS, In_2_S_3_, and ZnSe, alongside six familiar compounds, AlSb, CuGaSe_2_ (CGS), CuS, MoS_2_, Sb_2_S_3_, and WSe_2_, as the back surface field (BSF) layers. These heterostructures aim to uncover the potential of AgBiS_2_ in the realm of photovoltaic technology. When AgBiS_2_ functions within a singular heterojunction, specifically in configurations such as n-CdS/p-AgBiS_2_, n-In_2_S_3_/p-AgBiS_2_, and n-ZnSe/p-AgBiS_2_, the resulting values for open-circuit voltage (*V*_OC_) and the short circuit current (*J*_SC_) are found to be ∼0.90 V and ∼32 mA/cm^2^, respectively, while the corresponding power conversion efficiencies (PCE) are 23.56%, 22.60%, and 23.62%, respectively. On the contrary, the incorporation of various BSF layers like AlSb, CGS, CuS, MoS_2_, Sb_2_S_3_, and WSe_2_ results in a substantial increase in *V*_OC_, leading to an enhancement in PCE. Among the AgBiS_2_ based different dual-heterostructures, the outstanding PCE of 30.04% with a *V*_OC_ of 1.12 V is achieved by n-ZnSe/p-AgBiS_2_/p^+^-Sb_2_S_3_ device. In comparison, the n-ZnSe/p-AgBiS_2_/p^+^-CGS structure exhibits a similar PCE of 30.03% with a *V*_OC_ of 1.12 V. Additionally, the n-ZnSe/p-AgBiS_2_/p^+^-MoS_2_ arrangement demonstrates a PCE of 29.95% and a *V*_OC_ of 1.12 V. The effective band alignments observed at the interfaces of ZnSe/AgBiS_2_ and AgBiS_2_/MoS_2_, ZnSe/AgBiS_2_ and AgBiS_2_/CGS, as well as ZnSe/AgBiS_2_ and AgBiS_2_/Sb_2_S_3_ contribute to a substantial built-in potential, leading to an elevated *V*_OC_. As an alternative to ZnSe, the CdS window could offer similar performances, whereas In_2_S_3_ might provide a lower efficiency. The elaborate simulation findings highlight the substantial potential of AgBiS_2_ as an absorber, particularly when coupled with different windows and BSF layers. This opens avenues for experimental research focused on AgBiS_2_ in the era of photovoltaic cells.

## 1 Introduction

Leveraging the sun’s energy for generating electricity has emerged as a highly promising approach to address the world’s energy challenges. Yet, for solar power to be a competitive alternative to traditional energy sources, the technology responsible for converting sunlight into electricity, known as a solar cell, must strike a balance between reliability and cost-effectiveness. Researchers have explored various solar technologies, including wafer-based, thin-film-based, and organic-based technologies, to achieve these goals while maintaining high efficiency. Notably, crystalline silicon technology has made substantial strides, transitioning successfully from the laboratory to commercial integration, and now accounts for a significant portion, up to 90%, of the global photovoltaic market.^1^ Cost efficiency is evident when there is a reduction in material usage, coupled with an improvement in energy conversion efficiency. Wafer technology can achieve a high efficiency, while thin film technology excels in minimizing material usage. Achieving both of these objectives simultaneously is essential for enabling the cost-effective production of electricity and facilitating widespread market adoption of solar power.^2^

In recent years, thin-film solar cells (TFSCs) have garnered significant research interest due to their economical production costs, enhanced device flexibility, and remarkable stability combined with high efficiency. These qualities position them as a credible alternative to silicon-based photovoltaic (PV) systems.^3−6^ Several common types of thin-film solar cells including amorphous silicon (a-Si) solar cells have a conversion efficiency of 14.0%.^7^ Due to their inherent superiority over Si technologies in terms of temperature coefficient, energy production, and rate of degradation, cadmium telluride (CdTe)-based cells have become the most widely used thin-film photovoltaic technology. Worldwide installations of CdTe-based modules exceed 30 GW peak (GWp). Numerous businesses are producing these modules, which are being distributed at up to 18.6% efficiency, while lab cell efficiency exceeds 22%.^8^ Recently, NaSbS_2_ has also been considered as a promising photovoltaic absorber with a band gap of 1.59 eV.^9^ In addition, antimony selenosulfide, Sb_2_(S,Se)_3_ alloys have gained significant attention due to their capacity to adjust the optical bandgap energy with the PCE of 8.87%.^10^ Moreover, the copper indium gallium selenide (CIGS) TFSC is a novel technology that emerged in the late 1980s and has been confirmed in laboratory research to obtain an efficiency of more than 22%.^11^ The phase of industrialization has been attained by CdTe, CIS, and CIGS based TFSC; however, thin film panels based on CdTe include a significant quantity of hazardous cadmium, necessitating cautious management throughout their production and disposal phases. Concerns have arisen about production capacity limitations for CdTe and CIGS-based PV devices due to constraints in the supply of indium (In) and tellurium (Te). Addressing this challenge necessitates the development of alternative light absorber materials that are readily available and nontoxic.^12^

In this context, silver bismuth sulfide AgBiS_2_ (ABS) is a promising absorber candidate. ABS consists of silver, bismuth, and sulfur which are nontoxic and readily available elements. This makes it a more environmentally sustainable option compared to other traditional photovoltaic materials. It could be a hopeful absorber for PV applications due to its suitable direct optical bandgap of 1.2–1.3 eV and high absorption coefficient of 10^5^ cm^–1^.^13,14^ In addition, this compound has excellent thermal stability, a critical factor for ensuring the enduring dependability and efficiency of solar devices, particularly in the face of fluctuating external conditions. ABS crystallizes below its incongruent melting point of 801 ± 4 °C and can exhibit stability in both cubic and hexagonal phases. The hexagonal phase, known as matildite (β-AgBiS_2_), remains stable at temperatures below 195.15 ± 5 °C, while the cubic phase, schaphachite (α-AgBiS_2_), maintains stability in the temperature range of 195.15 ± 5–801 ± 4 °C.^13^ Furthermore, compounds based on bismuth may provide intriguing substitutes for substances that include lead. The metal bismuth is rather common in the crust of the planet; besides, Bi is a reasonably cheap and stable metal because it is a byproduct of the refinement of Pb, Cu, and Sn and has few major industrial applications.^15^ Several popular methods can be utilized to deposit ABS thin films including direct thermal evaporation, spray pyrolysis, vacuum fusing, flux techniques, polyol and microwave-assisted approaches, and hot-injection method.^16^ Eventually, AgBiS_2_ thin films may be produced by a facile and straightforward spin coating technique.^17^

There have previously been several studies on ABS solar cells. A previously documented PCE of 9.1% has been achieved with presynthesized colloidal ABS quantum dots, fabricated under inert conditions.^18^ In addition, the PCE of 6.4% has also been seen in ABS colloidal nanocrystal solar devices.^19^ Recently, solution-processed mixed ABS TFSC has demonstrated an efficiency of 7.3%.^20^ While a PCE of 26% has been found for ABS based PV cells through theoretical research.^21^ Unfortunately, despite ABS’s notable potential, not much research has been done, to the best of our knowledge, on it. Hence, in this study, a detailed simulation has been conducted based on ABS to explore its prospects. Besides, various kinds of window and BSF layers have been used with ABS to reveal the optimum performance.

Nevertheless, with a broader bandgap of 2.4 eV, the n-type CdS window facilitates the transmission of a significant portion of visible light.^22^ Consequently, it can be utilized as a window layer in conjunction with various absorber layers, including Si, CIGS, CdTe, and other materials.^23^ In a PV device, CdS exhibits superior and dominant characteristics as a semiconductor when utilized as a window layer material. Conversely, utilizing ZnSe as a window layer in lieu of CdS provides numerous benefits. These advantages encompass a 2.7 eV bandgap, facilitating widespread light transmission, and enhanced electron and hole mobilities.^24^ While molecular beam epitaxy is the conventional method, ZnSe can alternatively be produced through thermal evaporation,^25^ metal–organic chemical vapor deposition (CVD)^26^ and electrochemical deposition.^27^ Hence, ZnSe could serve as a commendable option for a window layer. However, numerous research groups have undertaken significant efforts to substitute the CdS window layer in thin film PV cells with an alternative semiconductor featuring a wide bandgap, primarily for environmental considerations. Indium sulfide (In_2_S_3_) has been identified as a promising alternative material owing to its stability, transparency, photoconductive properties, and the flexibility of its energy band gap.^28^ In_2_S_3_ is a binary semiconductor classified within the (III–VI) compound family. Its suitability stems from its nontoxic nature, outstanding optoelectric characteristics, impressive optical transparency, strong conductivity, and an optimal bandgap ranging from 2.1 to 2.9 eV.^29^

In this endeavor, aluminum antimonide (AlSb), copper gallium diselenide (CGS), copper sulfide (CuS), molybdenum disulfide (MoS_2_), antimony trisulfide (Sb_2_S_3_), and tungsten diselenide (WSe_2_) have been utilized as BSF layers due to their superior band alignment with ABS compound.

AlSb stands out as a compound with favorable optical and electrical properties, making it a viable alternative. Its optimal bandgap energy of 1.62 eV renders it effective in absorbing the solar spectrum.^30^ AlSb has already been employed as an absorber layer and has demonstrated impressive efficiency.^31^ Moreover, there is notable interest in semiconductor compounds derived from CuGaSe_2_, which exhibit crystallization in chalcopyrite structures.^32^ CGS exhibits characteristics of a p-type semiconductor, featuring a broad bandgap of 1.66 eV.^33^ The CGS compound has successfully demonstrated an experimental PCE ranging from 4.1 to 10% when utilized as an absorber layer.^34^ The flash evaporation, RF sputtering, laser-assisted evaporation, and electron beam evaporation methods can be used to grow the CGS layer.^34^ Additionally, in simulated investigations incorporating CuSbSe_2_ and CIGS absorber layers, CGS was used as a BSF layer. These investigations also assert its capability to imbibe longer wavelength photons, significantly enhancing short-circuit current.^33,35^

Materials like MoS_2_, WS_2_, MoSe_2_, WSe_2_, TiS_2_, TiSe_2_, etc. belong to the category of layered transition metal dichalcogenide semiconductor materials. These materials have gained significant attention as photovoltaic materials over the past few decades. Among them, MoS_2_ is noteworthy as an indirect bandgap material with a value of 1.3 eV. It transforms into a direct bandgap material with a value of 1.8 eV when it exists in a single atomic layer.^36^ The absorption coefficient of MoS_2_ is approximately 10^6^ cm^–^^1^, and this similarity holds across thicknesses ranging from a few micrometers to less than 100 Å.^37^ Moreover, the efficiency of MoS_2_ as an alternative material for absorber layers in TFSCs has already been explored and has been demonstrated to be highly promising. On the other hand, the remarkable properties of Sb_2_S_3_, such as its broad bandgap of 1.6–1.8 eV, significant absorption coefficient of 10^5^ cm^–1^, low melting point of 550 °C, inexpensive constituents, and low structural complexity with only one crystallographic phase make it an excellent candidate for use as a transparent absorber material.^38,39^ The advancement in crafting crystalline Sb_2_S_3_ thin films, whether employed as a buffer layer or as an absorber material in three-dimensional (3D) cells, has been investigated and exhibits significant positive outcomes.^40^ Moreover, WSe_2_ exhibits significant potential for the application of photoelectrochemical cells (PECs) and PV devices. WSe_2_ possesses a wide bandgap of 1.62 eV. It has demonstrated great promise and has already been employed as BSF in GeSe-based PV cells.^41^ Additionally, copper sulfide (CuS) holds significance as a crucial chalcogenide semiconductor, with its properties varying based on chemical composition. Gorai et al. have demonstrated that the solvent plays a pivotal role in controlling both the stoichiometry and morphology of copper sulfide crystals.^42^ CuS features a preferable bandgap of 1.55 eV for functioning the role of BSF layer.^43^

Therefore, in this article, ABS based novel double-heterostructure PV cells have been modeled and investigated thoroughly. This design has also investigated CdS, In_2_S_3_, and ZnSe as windows and AlSb, CuS, CGS, MoS_2_, Sb_2_S_3_, and WSe_2_ as BSF layers due to their superlative band alignment with ABS. This research stipulates here the optimal electron and hole transport layers for ABS solar device manufacturing in the future.

## 2 Proposed Structures and Numerical Computation

### 2.1 Designed Photovoltaic Devices

**Figure 1:**
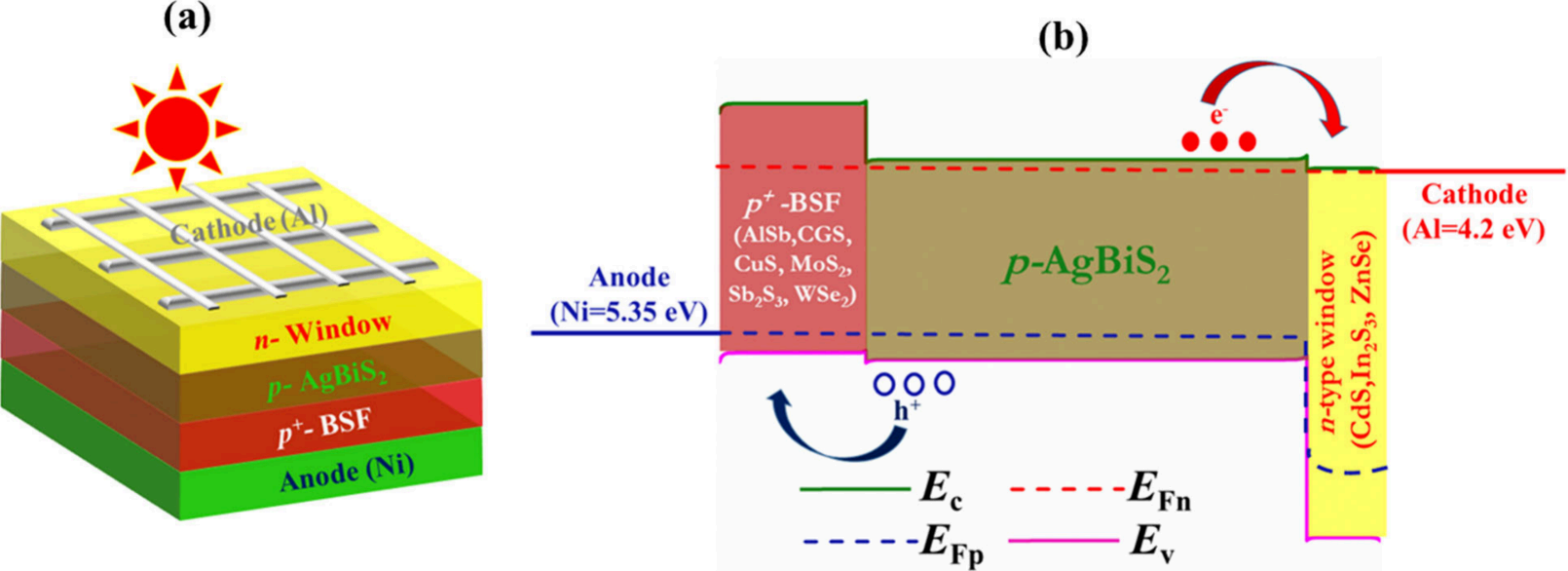
(a) Block architecture and (b) energy band layout under the lit condition of AgBiS_2_ based dual-heterostructure device.

Figure 1 illustrates both the (a) diagrammatic architecture and the (b) energy band layout of the illuminated state for the proposed ABS based TFSC. In this study, three window layers, namely, CdS, In_2_S_3_, and ZnSe are employed. For the BSF layer, six distinct compounds, AlSb, CGS, CuS, MoS_2_, Sb_2_S_3_, and WSe_2_ are utilized to assess the potentials of ABS.

In this context, photons traverse through the CdS, In_2_S_3_, and ZnSe window layers, where they are absorbed by ABS. Positioned beneath the absorber layer, the BSF layer is strategically placed to induce an additional depletion region. This outcome enhances the separation of electron–hole pairs, leading to the generation of a greater potential difference. The electron affinity, E.A. (χ), and ionization potential values for the CdS, In_2_S_3_, and ZnSe window layers are 4.4 and 6.8 eV, 4.4 and 6.75 eV, and 4.09 and 6.79 eV, respectively. The *E*_C_ and *E*_V_ for the ABS layer are 4.1 and 5.4 eV, respectively. On the flip side, the E.A. and ionization potential values for the AlSb, CGS, CuS, MoS_2_, Sb_2_S_3_, and WSe_2_ layers are 3.6 and 5.2 eV, 3.61 and 5.27 eV, 3.6 and 5.15 eV, 3.8 and 1.62 eV, 3.7 and 5.32 eV, and 3.62 and 5.24 eV, respectively. These values confirm that the band alignment of the heterostructures, using ABS as a p-type absorber and n-type window (CdS, In_2_S_3_, ZnSe) and p^+^-BSF (AlSb, CGS, CuS, MoS_2_, Sb_2_S_3_, WSe_2_), is appropriately configured. The dashed lines representing *E*_Fn_ and *E*_Fp_ in Figure 1(b) denote the quasi-Fermi levels for the electrons and holes, respectively. This figure further affirms that in the BSF layer, the *E*_Fn_ level is positioned above the valence band (VB) edge, whereas in the window layer, the *E*_Fp_ level is situated below the conduction band (CB) edge. As a result, within the ABS absorber, photogenerated carriers like electrons are directed toward the window (CdS, In_2_S_3_, ZnSe) and impeded by the BSF (AlSb, CGS, CuS, MoS_2_, Sb_2_S_3_, WSe_2_) layers. Conversely, holes migrate toward the BSF (AlSb, CGS, CuS, MoS_2_, Sb_2_S_3_, WSe_2_), encountering hindrance from the window (CdS, In_2_S_3_, ZnSe). The energy band layout has been shown using CdS window with ABS. The In_2_S_3_, and ZnSe window also provide similar alignment with the ABS. However, some spikes might be found in the ABS/BSF interfaces due to the variation of *E*_C_ and *E*_V_ values which are not shown in the figure. Aluminum (work function, ϕ = 4.2 eV) and nickel (ϕ = 5.35 eV) are employed as metal contacts for the cathode and anode, respectively.

### 2.2 Layers Properties and SCAPS Modeling

The simulation of the ABS based PV device was conducted comprehensively through one-dimensional solar cell capacitance (SCAPS-1D) software built by Burgelman et al.^44^ The simulation incorporated standard conditions, such as a single sun illumination intensity of 100 mW/cm^2^ and the AM 1.5G standard spectrum. In this simulation, a working temperature of 300 K was considered.

The thermal velocities utilized for the front and rear contacts for electrons and holes were 10^7^ and 10^5^ cm/s, respectively. The impact of altering depth, carrier, and defect density of each layer were examined. In conclusion, it was explored whether modifications to the window or BSF layer could significantly enhance the proposed structure. The input parameters for various window and absorber layers utilized in this simulation are presented in Table 1, while the input parameters for different BSF layers are detailed in Table 2. The effective densities of states for the conduction and valence bands are represented by *N*_C_ and *N*_V_, respectively. These values for the absorber layer were determined using the following formulas:^43^
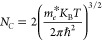
1
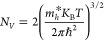
2In this context, *m*_e_^*^ and *m*_h_^*^ denote the effective masses of electrons and holes, respectively. *m*_e_^*^ and *m*_h_^*^ for ABS are 0.35 m_o_ and 0.722 m_o_ which were used to compute the *N*_C_ and *N*_V_.

**Table 1:** Input Parameters of ABS and Different Window Layers Used in This Investigation

Parameter	n-CdS^45^	n-In_2_S_3_^46^	n-ZnSe^47^	p-AgBiS_2_^48−52^
band gap, *E*_g_ (eV)	2.4	2.35	2.7	1.3
electron affinity, E.A. (χ) (eV)	4.4	4.40	4.09	4.1
dielectric permittivity, ε (relative)	10.0	13.50	10.0	11.50
CB effective density of states, *N*_c_ (cm^–3^)	2.2 × 10^18^	2.2 × 10^17^	1.5 × 10^18^	5.02 × 10^18^
VB effective density of states, *N*_v_ (cm^–3^)	1.8 × 10^19^	1.8 × 10^19^	1.8 × 10^18^	1.54 × 10^19^
electron mobility, μ_e_ (cm^2^ V^–1^ s^–1^)	100	100	50	7.00 × 10^–2^
hole mobility, μ_h_ (cm^2^ V^–1^ s^–1^)	25	25	20	3.20 × 10^–2^
shallow uniform donor density, *N*_D_ (1/cm^3^)	1.00 × 10^18^	1.00 × 10^17^	1.00 × 10^18^	0.00 × 10^0^
shallow uniform acceptor density, *N*_A_ (1/cm^3^)	1.00 × 10^7^	0.00 × 10^0^	0.00 × 10^0^	4.50 × 10^15^

**Table 2:** Input Parameters of Various BSF Layers Embarked on in This Simulation

Parameter	p^+^-AlSb^53^	p^+^-CuGaSe_2_^34^	p^+^-CuS^43^	p^+^-MoS_2_^54^	p^+^-Sb_2_S_3_^55^	p^+^-WSe_2_^41^
*E*_g_ (eV)	1.6	1.66	1.55	1.62	1.62	1.62
χ (eV)	3.6	3.61	3.6	3.80	3.7	3.62
ε (relative)	12.040	8.90	6.50	10.07	7.08	13.8
*N*_c_ (cm^–3^)	7.8 × 10^17^	2.2 × 10^17^	1.2 × 10^19^	2.80 × 10^19^	2.00 × 10^19^	8.3 × 10^18^
*N*_v_ (cm^–3^)	1.7 × 10^19^	1.8 × 10^18^	1.8 × 10^19^	1.0 × 10^19^	1.00 × 10^19^	1.6 × 10^19^
μ_e_ (cm^2^ V^–1^ s^–1^)	2.0 × 10^2^	100	12	1.2 × 10^1^	9.8	100
μ_h_ (cm^2^ V^–1^ s^–1^)	4.2 × 10^2^	250	9	2.8 × 10^0^	10	500
*N*_D_ (1/cm^3^)	0.00 × 10^0^	0.00 × 10^0^	0.00 × 10^0^	0.00 × 10^0^	0.00 × 10^0^	0.00 × 10^0^
*N*_A_ (1/cm^3^)	1.00 × 10^18^	1.00 × 10^18^	1.00 × 10^19^	1.00 × 10^19^	2.00 × 10^19^	1.00 × 10^18^

## 3 Results and Discussions

### 3.1 Light-Dependent Current–Voltage and Quantum Efficiency Characteristics of AgBiS_2_ PV Devices

**Figure 2:**
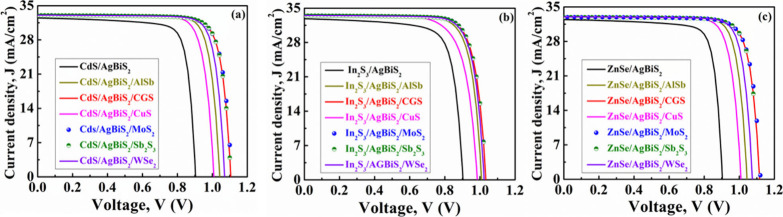
*J*-*V* graphs of AgBiS_2_ heterostructures employing diverse hole transport layers for (a) CdS, (b) In_2_S_3_, and (c) ZnSe window layers.

Figure 2(a) illustrates the current–voltage (*J*-*V*) characteristics among seven heterostructures in ABS-based solar devices using CdS as the window. According to this figure, a single heterojunction of n-CdS/p-ABS demonstrates the *J*_SC_ of 32.43 mA/cm^2^ and the *V*_OC_ of 0.90 V. Enhancements in performance can be achieved by incorporating different BSF layers. The *J*_SC_ undergoes a slight increase, reaching nearly 33.0 mA/cm^2^, as surface recombination diminishes for all the double-heterostructures.^56^ In the dual-heterostructure of n-CdS/p-ABS/p^+^-AlSb, the *V*_OC_ reaches to 1.04 V and the observed increase in *V*_OC_ of 0.14 V may be attributed to the generation of a built-in potential at the ABS/AlSb interface.^47,56^ Maximum *V*_OC_ of 1.10 V is reached by incorporating CGS, MoS_2_, and Sb_2_S_3_ as BSF layers. The higher *V*_OC_ is triggered by the elevated built-in potential developed in the ABS/CGS, ABS/MoS_2_, and ABS/Sb_2_S_3_ interfaces as a result of their precise band alignment.^47,56^ However, CdS/ABS/CuS, CdS/ABS/AlSb, and CdS/ABS/WeS_2_ devices provide the *V*_OC_ of 1.0, 1.04, and 1.07 V, respectively

Figure 2(b) showcases the *J*-*V* curves across seven heterostructures of ABS-based devices using In_2_S_3_ as a window. As indicated in this figure, a single heterojunction of n-In_2_S_3_/p-ABS exhibits a *J*_SC_ of 32.68 mA/cm^2^ and a *V*_OC_ of 0.90 V. Further performance augmentations are realized by introducing various BSF layers. The *J*_SC_ rises to 33.50 mA/cm^2^ for all arrangements due to the decrease in surface recombination velocity, but the *V*_OC_ varies for different structures due to the different band alignment. The maximum *V*_OC_ of 1.04 V has been observed in n-In_2_S_3_/p-ABS/p^+^-MoS_2_, n-In_2_S_3_/p-ABS/p^+^-CGS, and n-In_2_S_3_/p-ABS/p^+^-Sb_2_S_3_ devices. AlSb, WSe_2_, and CuS have been found to exhibit *V*_OC_s of 1.0, 1.02, and 0.99 V, respectively, when taken into consideration as BSF.

Figure 2(c) demonstrates the *J*-*V* performance of ABS-based PV devices, comparing configurations with and without AlSb, CGS, CuS, MoS_2_, Sb_2_S_3_, and WSe_2_ as hole transport layers, while utilizing ZnSe as the window. In this figure, it is noted that akin to the CdS and In_2_S_3_ window layers, *V*_OC_ shows an uptick with the inclusion of different BSF layers. The n-ZnSe/p-ABS configuration yields a *V*_OC_ of around 0.90 V with a *J*_SC_ of 32.26 mA/cm^2^. This *J*_SC_ has improves to a nearly 33.0 mA/cm^2^ due to the addition of various BSF layers. The maximum *V*_OC_ of 1.12 V has been acquired when MoS_2_, CGS, and Sb_2_S_3_ have been used as the BSF layer. Besides, 1.07, 1.04, and 1.01 V were obtained by using WSe_2_, AlSb, and CuS BSF layers, respectively.

**Figure 3:**
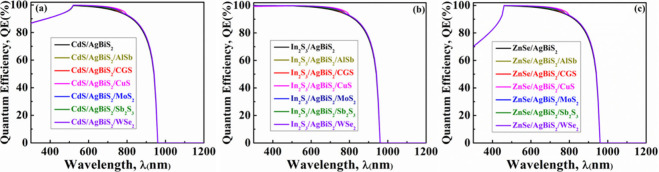
QE graphs of AgBiS_2_ heterostructures employing diverse hole transport layers for (a) CdS, (b) In_2_S_3_, and (c) ZnSe window layers.

The quantum efficiency (QE) is dependent on the light wavelength (λ) and can be characterized as the ratio of charge carriers generated by a solar cell to the number of incident photons striking the cell.^47^ In Figure 3, the QE is depicted for the modeled ABS-based structures. It is clear that the QE, both without and with different BSF layers, remains entirely similar when considering distinct window layers: CdS in Figure 3(a), In_2_S_3_ in Figure 3(b), and ZnSe in Figure 3(c). The QE begins to decline beyond 900 nm as the photon energy (*h*υ) becomes lower than the bandgap (*E*_g_) energy of the ABS absorber, eventually reaching zero.^47^ However, it is seen that in the shorter wavelength region (300–500 nm), In_2_S_3_ window-based ABS solar devices provide higher QE compared to CdS and ZnSe. Nevertheless, their *J*_SC_ performances are almost similar. Light creates a significant portion of the electron–hole pairs in the In_2_S_3_ region. However, the depletion zone’s electric field is unable to effectively separate these pairs. As a result, these pairs will recombine without contributing to the photocurrent. As a result, reverse saturation current rises lowering the *V*_OC_ as well. Because of this, compared to CdS or ZnSe, the ABS devices with In_2_S_3_ window offers lesser *V*_OC_.^57^

### 3.2 Function of the AgBiS_2_ Absorber Layer

This section provides results elucidating the influence of absorber layer thickness, acceptor density, and defect levels in ABS on key photovoltaic parameters including *J*_SC_, *V*_OC_, fill factor (FF), and PCE. The investigation encompasses various window such as CdS, In_2_S_3_, and ZnSe, coupled with a range of BSF layers including AlSb, CGS, CuS, MoS_2_, Sb_2_S_3_, and WSe_2_.

#### 3.2.1 Function of ABS Layer with CdS as Window and Various BSFs

**Figure 4:**
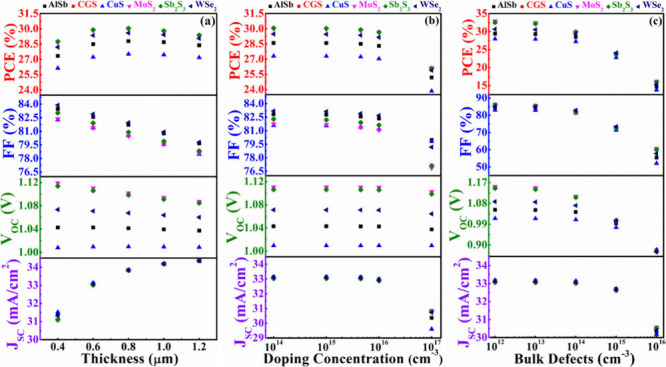
PV performances of AgBiS_2_ solar device using CdS as window and various BSF layers in conformity with (a) width, (b) carrier level, and (c) bulk flaws of absorber layer.

This segment presents empirical findings highlighting the impact of the thickness, acceptor, and defect level of the ABS absorber layer on *J*_SC_, *V*_OC_, FF, and PCE. This analysis considers CdS as the window, along with diverse BSF layers such as AlSb, CGS, CuS, MoS_2_, Sb_2_S_3_, and WSe_2_. In Figure 4(a), the graph illustrates the performance variation of the cell concerning the ABS layer thickness with CdS as the window layer. The depth ranges from 0.4 to 1.2 μm to determine the optimal performance of the proposed structure. The figure reveals that *J*_SC_ increases from 31.00 to 34.50 mA/cm^2^ within this range for all structures. This rise in current is attributed to the thicker absorber layer absorbing more photons, generating an increased number of electron–hole pairs.^58^ However, for CuS, AlSb, and WSe_2_, the *V*_OC_ demonstrates almost independent behavior with varying thickness of ABS. In contrast, for CGS, MoS_2_, and Sb_2_S_3_, the *V*_OC_ experiences a certain decrease with the increase in thickness. The *V*_OC_ diminishes as recombination grows with a thicker absorber, raising the dark current in the process. The FF exhibits a nearly continuous fall for all devices, declining from 84.10% to 78.00%, as the absorber thickness upsurges. This decline in the FF is attributed to its inverse relationship with thickness, primarily caused by an elevated series resistance. With a notable increase in the *J*_SC_, the PCE experiences a gradual ascent for all cases. The PCE reaches its maximum when the thickness is 0.8 μm. The structures based on the BSF layer of CGS and Sb_2_S_3_ had the highest PCE, 30.05%. In addition, the CdS/ABS/MoS_2_ device also delivers a similar PCE of 29.99% at a 0.8 mm thick absorber. However, with a further increase in thickness from 0.8 to 1.2 μm, the PCE shows a slight decrease.

Figure 4(b) illustrates the impact of the acceptor level across a range of 10^14^–10^17^ cm^–3^ of ABS and CdS as the window layer while maintaining a constant width of 0.6 μm. The *V*_OC_ is observed to remain constant for all cases with varying carrier density of ABS. The *J*_SC_ is observed to diminution at carrier level exceeding 10^17^ cm^–3^. At carrier concentrations exceeding 10^17^ cm^–3^, the *J*_SC_ for all hetero devices diminishes due to the free carrier recombination. Moreover, an elevated acceptor concentration leads to heightened carrier recombination in the bulk region, consistent with observations in a preceding report.^58^ The FF and PCE also decline at carrier densities surpassing 10^17^ cm^–3^ and beyond. The PCE follows the *J*_SC_ and FF, and the FF lowers as a result of the ideality factor for all devices expanding.^46^

Figure 4(c) illustrates the impact of bulk defects, *N*_t_, in the ABS absorber material on device performance. The study considers a range of *N*_t_ values spanning from 10^12^ cm^–3^ to 10^16^ cm^–3^, while maintaining a constant width of 0.6 μm and acceptor density of 4.5 × 10^15^ cm^–3^. The graph clearly indicates the consistent behavior of all PV parameters for all structures at low defect concentrations. However, as defects grow beyond 10^14^ cm^–3^, all parameters exhibit a decline. Specifically, the *J*_SC_ remains stable at all heterostructures between 10^12^ and 10^15^ cm^–3^ but experiences a sudden drop at 10^16^ cm^–3^. Because more imperfections prevent photons from being absorbed, the *J*_SC_ lowers.^34^ The *V*_OC_ of the all solar devices shows a significant lessening with defects, the progression of the defects boosts Shockley–Read–Hall (SRH) recombination which lowers the voltage.^34^ The ideality factor’s elevation at higher defect levels leads to a dramatic reduction in the FF, dropping from 88.88% to 50.90%.^54^ Consequently, based on *J*_SC_, *V*_OC_, and FF, the PCE also experiences a decline for all cases of ABS based PV devices within the range of defects.

#### 3.2.2 Function of Absorber Layer with In_2_S_3_ as Window and Various BSFs

**Figure 5:**
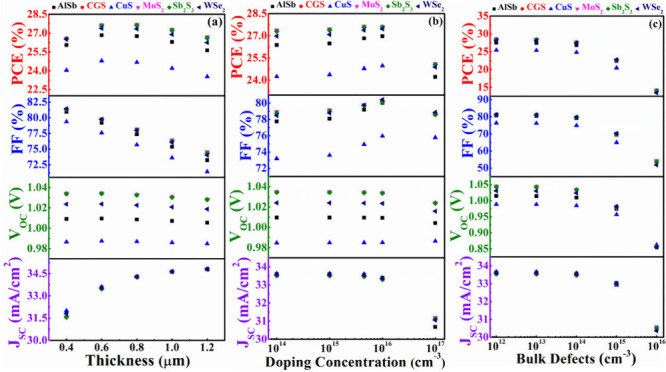
PV performances of AgBiS_2_ solar device using In_2_S_3_ as window and various BSF layers in conformity with (a) width, (b) carrier level, and (c) bulk flaws of absorber layer.

In this section, observational results are presented that underscore the influence of the absorber layer thickness, acceptor density, and defect level on *J*_SC_, *V*_OC_, FF, and PCE. This analysis specifically incorporates In_2_S_3_ as the window layer, accompanied by a variety of BSF layers, including AlSb, CGS, CuS, MoS_2_, Sb_2_S_3_, and WSe_2_. Figure 5(a) illustrates the photovoltaic performance of the ABS solar device utilizing In_2_S_3_ as the window and various BSF layers. The graph illustrates a growth in the *J*_SC_ from 31.50 to 34.55 mA/cm^2^ across the 0.4–1.2 μm width range for all cases. This escalation in current is linked to the thicker absorber layer, which effectively absorbs more photons, leading to the generation of an augmented number of electron–hole pairs.^54^ From Figure 5(a), it is also evident that the maximum *J*_SC_ occurs at a thickness of 1.2 μm. The *V*_OC_ remains consistent as the thickness upturns. However, among the structures studied, the n-In_2_S_3_/p-ABS/p^+^-CuS configuration exhibits the lowest *V*_OC_, whereas the n-In_2_S_3_/p-ABS/p^+^-Sb_2_S_3_ or MoS_2_ or CGS structures demonstrates the highest *V*_OC_. The FF undergoes a gradual decline for the all designed structures by virtue of the series resistance with the width of ABS. The peak PCE is achieved at a thickness of 0.8 μm. Nevertheless, beyond this point, with a subsequent increase in thickness from 0.8 to 1.2 μm, all of the heterostructures show a marginal decline in PCE. The PCE values are closely comparable for all structures, except for the n-In_2_S_3_/p-ABS/p^+^-CuS.

Figure 5(b) demonstrates the discrepancy in photovoltaic parameters kin to the acceptor density (ranging from 10^14^ to 10^17^ cm^–3^) of ABS, while keeping a consistent width of 0.6 μm. This figure indicates that the *V*_OC_ for all devices remains unaffected by the doping level of ABS up to 10^16^ cm^–3^. Then, the *V*_OC_ drops except for the CuS BSF layer. As doping density rises, auger recombination takes center stage and growths the energy exchanges between impurities and carriers. This mechanism along with carrier loss may cause a reduction in *V*_OC_.^59^ The *J*_SC_ for all cases remains relatively stable up to a doping of 10^16^ cm^–3^, but it experiences a sudden drop at 10^17^ cm^–3^. The higher doping raises the free carrier recombination which attributes lower current.^58^ The FF and PCE for all PV cells gradually rise with the acceptor level and then abruptly drop at 1 × 10^17^ cm^–3^. The variation of diode parameters is ascribed to this result.^60^ Although the PCE is notably low for the n-In_2_S_3_/p-ABS/p^+^-AlSb device at a doping concentration of 10^17^ cm^–3^.

Figure 5(c) demonstrates the influence of *N*_t_ in the ABS absorber material on the device’s performance. The investigation encompasses a range of *N*_t_ values of 10^12^–10^16^ cm^–3^, with a constant thickness of 0.6 μm and acceptor density (*N*_A_) of 4.5 × 10^15^ cm^–3^. The graph clearly illustrates the consistent behavior among all PV parameters at low defects. However, as defects surpass 10^14^ cm^–3^, there is a notable decline in all parameters. Specifically, *J*_SC_ remains stable within the range of 10^12^–10^14^ cm^–3^ but experiences a sudden drop at 10^16^ cm^–3^ for all heterostructures. The *V*_OC_ of all the cells exhibits a significant diminution with defects, attributed to SRH recombination.^34^ The ideality factor’s rise at higher defect levels leads to a substantial reduction in the FF, decreasing from 81.55% to 51.00%.^54^ Consequently, with respect to *J*_SC_, *V*_OC_, and FF, the PCE also undergoes a decline within the range of defects. The In_2_S_3_ window layer exhibits a diminished performance when paired with the ABS absorber, primarily due to lower *V*_OC_ and FF values compared to CdS and ZnSe.

#### 3.2.3 Function of Absorber Layer with ZnSe as Window and Various BSFs

**Figure 6:**
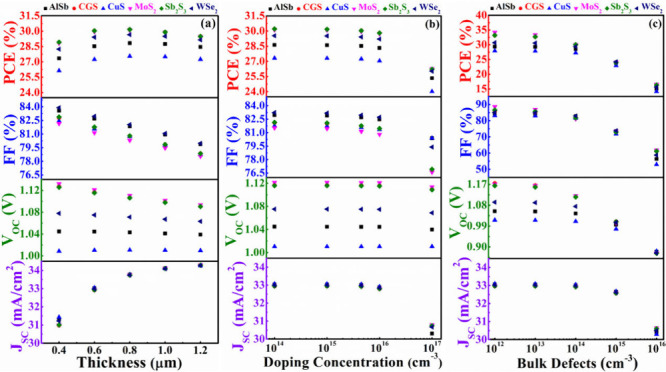
PV performances of AgBiS_2_ solar devices using ZnSe as window and various BSF layers in conformity with (a) width, (b) carrier level, and (c) bulk flaws of absorber layer.

Figure 6 depicts the PV characteristics of the ABS solar device, incorporating ZnSe as the window layer along with diverse BSF layers. The examination explores the impact of absorber layer depth, acceptor level, and bulk defects on the device’s performance. Figure 6(a) reveals a continuous augmentation of *J*_SC_ as the thickness grows in the case of all BSF layers. Notably, the *J*_SC_ demonstrates a nearly uninterrupted escalation from ∼27 to 30 mA/cm^2^ across various BSF layers. Because of the larger absorber layer’s ability to take in more photons and produce more electron–hole pairs, there has been a rise in current.^58^ The *V*_OC_ exhibits stability as depth upturns when employing WSe_2_, AlSb, and CuS as BSF layers. Besides, the structure n-ZnSe/p-ABS with CGS or MoS_2_ or Sb_2_S_3_ BSF attains a peak *V*_OC_ at a thickness of 0.4 μm. However, beyond this point, *V*_OC_ experiences a decline as width rises for these particular structures as like In_2_S_3_ window. The FF consistently diminishes as the width rises due to the escalation of the series resistance. Additionally, the figure illustrates that the PCE reaches its maximum of 30% for the Sb_2_S_3_, CGS, and MoS_2_ BSF layer-based structures.

Figure 6(b) delineates the changes in PV parameters concerning the doping density of ABS, spanning from 10^14^ cm^–3^ to 10^17^ cm^–3^, while maintaining a constant width of 0.6 μm. The *J*_SC_ for all heterostructures maintains stability up to a doping concentration of 1 × 10^16^ cm^–3^ but undergoes a sudden decrease at 1 × 10^17^ cm^–3^. Higher doping results in more free carrier recombination, which reduces current.^58^ The FF experiences a significant decline at the maximum doping concentration due to the variation of diode parameters.^54^ The *V*_OC_ remains nearly constant with an increase in concentration, and akin to the thickness variation illustration, the maximum *V*_OC_ is achieved when MoS_2_ is utilized as the BSF. The PCE remains relatively constant up to 10^16^ cm^–3^ but exhibits a sudden drop at higher concentrations, depending on FF and *J*_SC_.

Figure 6(c) illustrates the impact of *N*_t_ within the ABS absorber material on the device’s performance. The examination covers a spectrum of *N*_t_ values (10^12^–10^16^ cm^–3^), maintaining a consistent thickness of 0.6 μm and a fixed *N*_A_ at 4.5 × 10^15^ cm^–3^. The graph vividly demonstrates uniform trends across all PV parameters at low defect concentrations. However, once defects exceed 10^14^ cm^–3^, there is a noticeable decline in all parameters for the proposed devices. Due to SRH recombination, all of the cells’ *V*_OC_ shows a marked reduction with bulk faults.^34^ The FF of all cases shrinks substantially as one reaches higher defect levels due to the ideality factor’s growth.^59^ Moreover, higher bulk flaws make it more difficult to gather photons; hence, it makes sense for all structures’ *J*_SC_ to go down.^61^ Consequently, the PCE sharply declines with high flaws as a result of *J*_SC_, *V*_OC_, and FF degradation in every scenario.

### 3.3 Function of Different Window Layers

#### 3.3.1 CdS Window Layer Impacts with Various BSFs

**Figure 7:**
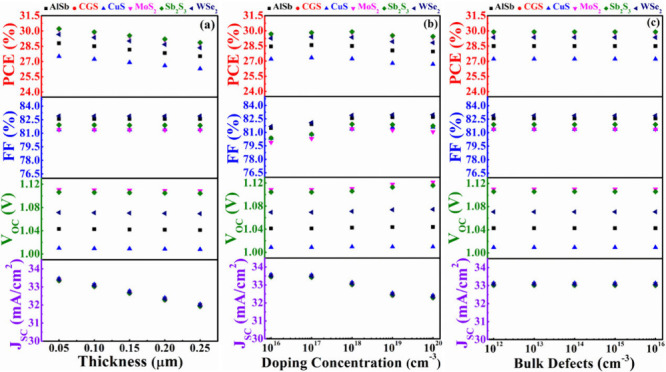
PV performances of AgBiS_2_ solar devices using CdS as window and various BSF layers in conformity with (a) width, (b) carrier level, and (c) bulk flaws of CdS.

In this context, the impact of a CdS window layer has been investigated when ABS serves as the absorber, considering AlSb, CGS, CuS, MoS_2_, Sb_2_S_3_, and WSe_2_ as the BSF layers. Figures 7(a)-(c) illustrate the variations in PV parameters concerning the depth, donor concentration, and flaw levels, respectively, of the CdS. The width, doping concentration, and bulk flaws of CdS are varied from 0.05 to 0.25 μm, from 10^16^ to 10^20^ cm^–3^, and from 10^12^ to 10^16^ cm^–3^, respectively, to examine the effects of the window layer. In Figure 7(a), the *V*_OC_, FF exhibits a closely persistent trend with the width of the CdS, while the *J*_SC_ and PCE slightly decrease with an increase in thickness for all hetero devices. Efficiency declines as a result of the thicker CdS boosting the minority charge carrier and recombination rates.^54^

Similarly, Figure 7(b) displays that the donor levels of CdS have fiddling dominance over all of the cell performances. PV characteristics continue to be independent of CdS layer defect levels, as further evidenced by Figure 7(c). The carrier diffusion length and lifetime show insignificant changes with the variations in depth, carrier level, and bulk flaws of CdS within the investigated ranges, resulting in the cell performance demonstrating an almost constant nature.^58^ The findings from Figures 7(a-c) reveal that structures n-CdS/p-ABS/p^+^-MoS_2_ and n-CdS/p-ABS/p^+^-Sb_2_S_3_ consistently yield the highest PCE, even when altering the depth, doping, and bulk defects of the CdS window layer. The structure n-CdS/p-ABS/p^+^-CGS also closely approaches the maximum output observed in these structures.

#### 3.3.2 In_2_S_3_ Window Layer Impacts with Various BSFs

**Figure 8:**
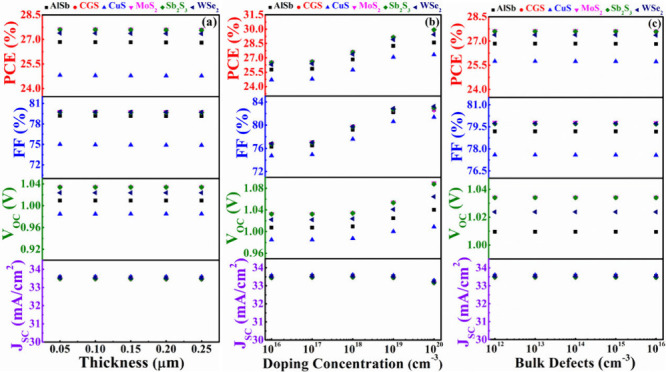
PV performances of AgBiS_2_ solar devices using In_2_S_3_ as window and various BSF layers in conformity with (a) width (b) carrier level, and (c) bulk flaws of In_2_S_3_.

In this scenario, the influence of an In_2_S_3_ window has been explored with ABS as the absorber, while considering AlSb, CGS, CuS, MoS_2_, Sb_2_S_3_, and WSe_2_ BSF layers. Figure 8(a)-(c) depicts the variations in PV parameters concerning the width, donor levels, and bulk defects of the In_2_S_3_. The depth, carrier, and defects of In_2_S_3_ are systematically varied (0.05–0.25 μm), (10^16^–10^20^ cm^–3^), and (10^12^–10^16^ cm^–3^), respectively, to assess the impacts of the In_2_S_3_. In Figure 8(a), the *J*_SC_, *V*_OC_, FF, and PCE of all proposed devices exhibit consistently constant behavior with changes in the width of the In_2_S_3_ layer. However, in Figure 8(b), it is evident that PCE, FF, and *V*_OC_ trend up with higher doping density, while *J*_SC_ remains nearly unchanged. The built-in potential might be elevated and series resistance mitigates with donor density responsible to raising the *V*_OC_ and FF, respectively.^56^Figure 8(c) also presents that the *J*_SC_, *V*_OC_, FF, and PCE of all proposed devices demonstrate a consistently stable response when the defects of the In_2_S_3_ layer is altered.

#### 3.3.3 ZnSe Window Layer Impacts with Various BSFs

**Figure 9:**
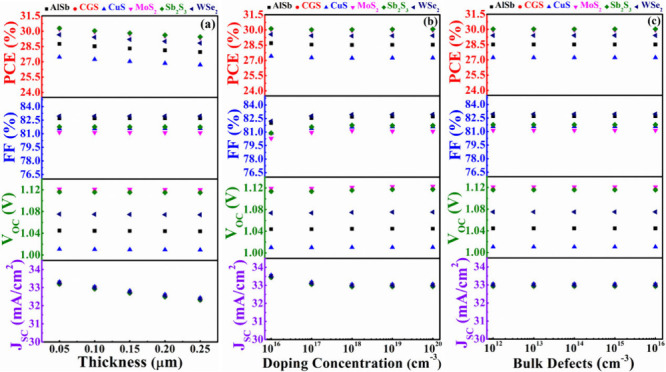
PV performances of AgBiS_2_ solar devices using ZnSe as window and various BSF layers in conformity with (a) width, (b) carrier level, and (c) bulk flaws of ZnSe.

In this context, the impact of a ZnSe window layer has been investigated with ABS as the absorber by considering various BSF layers. Figure 9(a)-(c) illustrates the variations in PV parameters concerning the depth, doping concentration, and flaw levels of the ZnSe. The depth, doping, and defects of ZnSe were systematically varied within the ranges of 0.05–0.25 μm, 10^16^–10^20^ cm^–3^, and 10^12^–10^16^ cm^–3^, respectively, to assess the impacts of the ZnSe. In Figure 9(a-c), the *J*_SC_, *V*_OC_, FF, and PCE consistently display perpetual behavior with changes in the width, doping, and defect of the ZnSe layer. Maximum performances have been obtained from the structures of n-ZnSe/p-ABS/p^+^-MoS_2_, n-ZnSe/p-ABS/p^+^-Sb_2_S_3_, and n-ZnSe/p-ABS/p^+^-CGS, similar to the CdS window discussed in section 3.3.1.

### 3.4 Functions of Different BSF Layers

The impact of different BSF layers on the ABS-based PV device was explored and analyzed through variations in thickness, acceptor density, and defect levels. The thickness, doping, and defects of six BSF layers are methodically altered within the ranges of 0.1–0.5 μm, 10^16^–10^20^ cm^–3^, and 10^12^–10^16^ cm^–3^, respectively, to evaluate the effects of the BSF layers. This investigation includes three distinct window layers: CdS, In_2_S_3_, and ZnSe.

#### 3.4.1 Functions of Different BSF Layers with CdS Window

**Figure 10:**
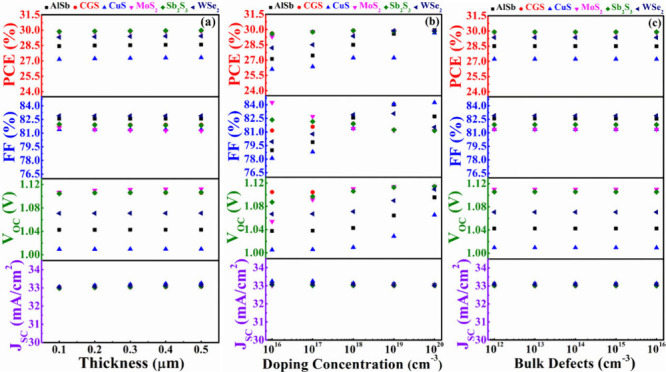
PV performances of AgBiS_2_ solar devices using CdS as window and various BSF layers in conformity with (a) width, (b) carrier level, and (c) bulk flaws of BSFs.

Figure 10(a) illustrates the correlation between the width of the AlSb, CGS, CuS, MoS_2_, Sb_2_S_3_, and WSe_2_ BSF layers and the corresponding output parameters with CdS as the window layer. The width of the different BSF layers is varied within the range of 0.1–0.5 μm. The PV parameters consistently demonstrate a constant behavior with variations in the width of six BSF layers. In this figure, it is observed that MoS_2_ as the BSF layer yields the highest *V*_OC_, and Sb_2_S_3_ as the BSF results in the maximum PCE. CGS, serving as a BSF, closely aligns with the output parameters of the MoS_2_ and Sb_2_S_3_ BSF layers.

Figure 10(b) illustrates the impact of varying acceptor concentrations for six different BSFs on output parameters, showing fluctuations between 10^16^ and 10^20^ cm^–3^. The figure assures that within the specified range of acceptor level, *J*_SC_ exhibits almost uniform behavior for all BSF layers. However, the *V*_OC_ of each device is increased with the doping of all BSF layers. The *V*_OC_ is likewise raised by a shift in built-in potential with the acceptor concentration of BSF layers.^56^ For AlSb, MoS_2_, Sb_2_S_3_, and WSe_2_ BSF structures, the surge in nonradiative recombination causes the FF to fall at a high carrier level.^59^ Conversely, the FF stays constant for CGS, but for CuS BSF, it climbs with the carrier. The maximum FF of ∼84% has been obtained for the CdS/ABS/CuS structure from the 10^19^ cm^–3^ doping level. The PCE shows a constant trend for Sb_2_S_3_, CGS, and MoS_2_ BSF layer-contained devices. Nonetheless, the PCEs of CdS/ABS/AlSb, CdS/ABS/WSe_2_, and CdS/ABS/CuS rise with acceptor level of hole transport layer depending on *V*_OC_ and FF. It is also observed that at high doping concentrations of 10^19^ cm^–3^, the CuS, AlSb, and WSe_2_ contained structures also perform similarly to Sb_2_S_3_, MoS_2_, and CGS.

Figure 10(c) illustrates the PV performance based on the defect level of various BSF layers. The bulk defect level was varied from 10^12^ to 10^16^ cm^–3^ to assess its impact. This figure unequivocally demonstrates that the defects in six BSF layers have no discernible effect on the performances of the modeled ABS-based solar devices. After all, it can be concluded that Sb_2_S_3_, MoS_2_, and CGS have better ability as BSF layers to provide maximum efficiency of 30% for CdS/ABS based solar devices.

#### 3.4.2 Functions of Different BSF Layers with In_2_S_3_ Window

**Figure 11:**
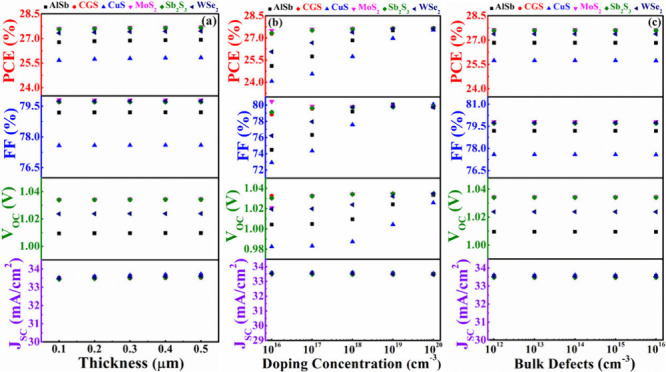
PV performances of AgBiS_2_ solar devices using In_2_S_3_ as a window and various BSF layers in conformity with (a) width, (b) carrier level, and (c) bulk flaws of BSFs.

In this context, the influence of different BSF layers has been explored, taking In_2_S_3_ as the window layer into consideration. Figure 11(a)-(c) depicts the changes in PV parameters in relation to the depth, doping, and bulk flaw levels of the different BSF layers. In Figure 11(a), every proposed structure steadily displays persistent behavior in accordance with the depth of diverse BSFs. However, in Figure 11(b), it is noticeable that the *J*_SC_ does not depend on the carrier density of any BSF layers. Due to a boost in built-in potential, the *V*_OC_ rises with each BSF layer in proportion to carrier density.^56^ While CGS, Sb_2_S_3_, and MoS_2_ offer the highest *V*_OC_ at low acceptor levels, all BSF can maximize *V*_OC_ at high levels. Despite the FF growth for AlSb, CuS, and WSe_2_ BSF layers with acceptor level, it stays unchanged for CGS, Sb_2_S_3_, and MoS_2_ hold devices. The PCE also follows the inclination of FF. It is noted that, at high doping level (10^19^ cm^–3^) of BSF, each structure delivers the maximum PCE 27% using the In_2_S_3_ window. Each proposed structure has a uniform response with defect density of various BSFs as shown in Figure 11(c). As compared to other BSF layers, the In_2_S_3_/ABS/CGS, In_2_S_3_/ABS/Sb_2_S_3_, and In_2_S_3_/ABS/MoS_2_ heterostructures give the best overall results. However, because of the lesser FF, In_2_S_3_ window-based devices might offer lower PCE than CdS or ZnSe. The decline in performance of In_2_S_3_-based devices may be due to a boost in ideality factor.^59^

#### 3.4.3 Functions of Different BSF Layers with ZnSe Window

**Figure 12:**
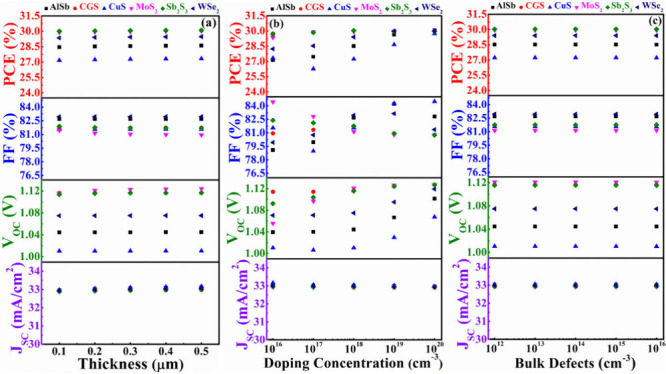
PV performances of AgBiS_2_ solar devices using ZnSe as a window and various BSF layers in conformity with (a) width, (b) carrier level, and (c) bulk flaws of BSFs.

In this particular investigation, the impression of different BSF layers has been delved into, with ZnSe serving as the window layer. Figure 12(a)-(c) illustrates the variations in PV parameters concerning the width, doping, and bulk flaw levels of these diverse BSF layers. In Figure 12(a), the *J*_SC_, *V*_OC_, FF, and PCE of every structure consistently exhibit a constant behavior with the depth of diverse BSF. However, in Figure 12(b), it is noteworthy that the *V*_OC_ moves upward for all BSF layers with carrier level due to the rise in built-in potential.^56^ Notably, the ZnSe window along with MoS_2_, CGS, and Sb_2_S_3_ BSF have been found to yield the maximum *V*_OC_ for ABS based devices. The main cause of this high *V*_OC_ value is the appropriate band alignment between the window and absorber. Furthermore, because nonradiative recombination is growing, the FF for all cases, aside from CuS, degrades alarmingly with high carriers.^59^ While the efficiency of the others nearly stays constant with acceptor density, it improves for CuS, WSe_2_, and AlSb BSF depend on FF and *V*_OC_. Each structure’s *J*_SC_, *V*_OC_, FF, and PCE continuously display a steady behavior in Figure 12(c) concerning the defects of BSFs. In the end, it can be said that Sb_2_S_3_, MoS_2_, and CGS are more suited to act as BSF layers in order to maximize the efficiency of 30% of solar systems based on ZnSe/ABS.

### 3.5 *C*–*V* Analysis of Designed Structures

**Figure 13:**
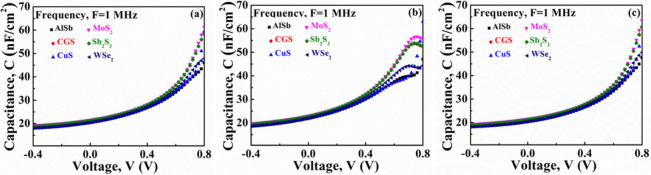
*C*–*V* characteristics of AgBiS_2_ PV heterostructures using various hole transport layers and (a) CdS, (b) In_2_S_3_, and (c) ZnSe window layers.

In Figure 13, the effect of capacitance is depicted within the voltage range of −0.4 to 0.8 V, alongside a consistent frequency of 1 MHz. This representation pertains to six distinct configurations of ABS-based photovoltaic devices, each incorporating one of three window layers: CdS, In_2_S_3_, and ZnSe. As illustrated in Figure 13(a-c), the capacitance experiences an exponential increase with the rise in the supplied voltage until reaching the saturation point. As the voltage increases from −0.4 V, there is a tendency for the capacitance of the specifications to rise. In the absence of bias, the PV cell undergoes a depletion situation. However, upon applying an upward bias of approximately 0.5 V, the width of the depletion region contracts, nearly matching the thickness of the absorber layer.

Consequently, an elevation in forward bias voltage leads to an increased capacitance while preserving the Mott–Schottky (MS) relationship.^62^ For CdS window-based structures, Figure 13(a) illustrates a notable increase in the capacitance for the MoS_2_, Sb_2_S_3_, and CGS BSF layers as the voltage rises, surpassing the capacitance levels observed in other BSF configurations. In Figure 13(b) for In_2_S_3_ window-based devices, it is evident that at lower voltages, the capacitance rises with the applied voltage, reaching a peak. Subsequently, as the voltage continues to increase, the capacitance starts to decrease. Notably, at lower voltages, the current remained significantly below the saturation current of the contact diode. Conversely, at higher voltages, the current is constrained to the saturation current at the contact,^63^ resulting in additional reduction in the voltage across the given structure. The ZnSe window-based cells provide a *C*–*V* response similar to that of CdS, as depicted in Figure 13(c).

The built-in potential (ψ_bi_) of each heterostructure has been estimated using the following Mott–Schottky (MS) relationship:
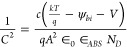
3where ∈_0_∈_ABS_ denotes permittivity, *V* specifies voltage, and *A* stands for the area of the diode.

**Figure 14:**
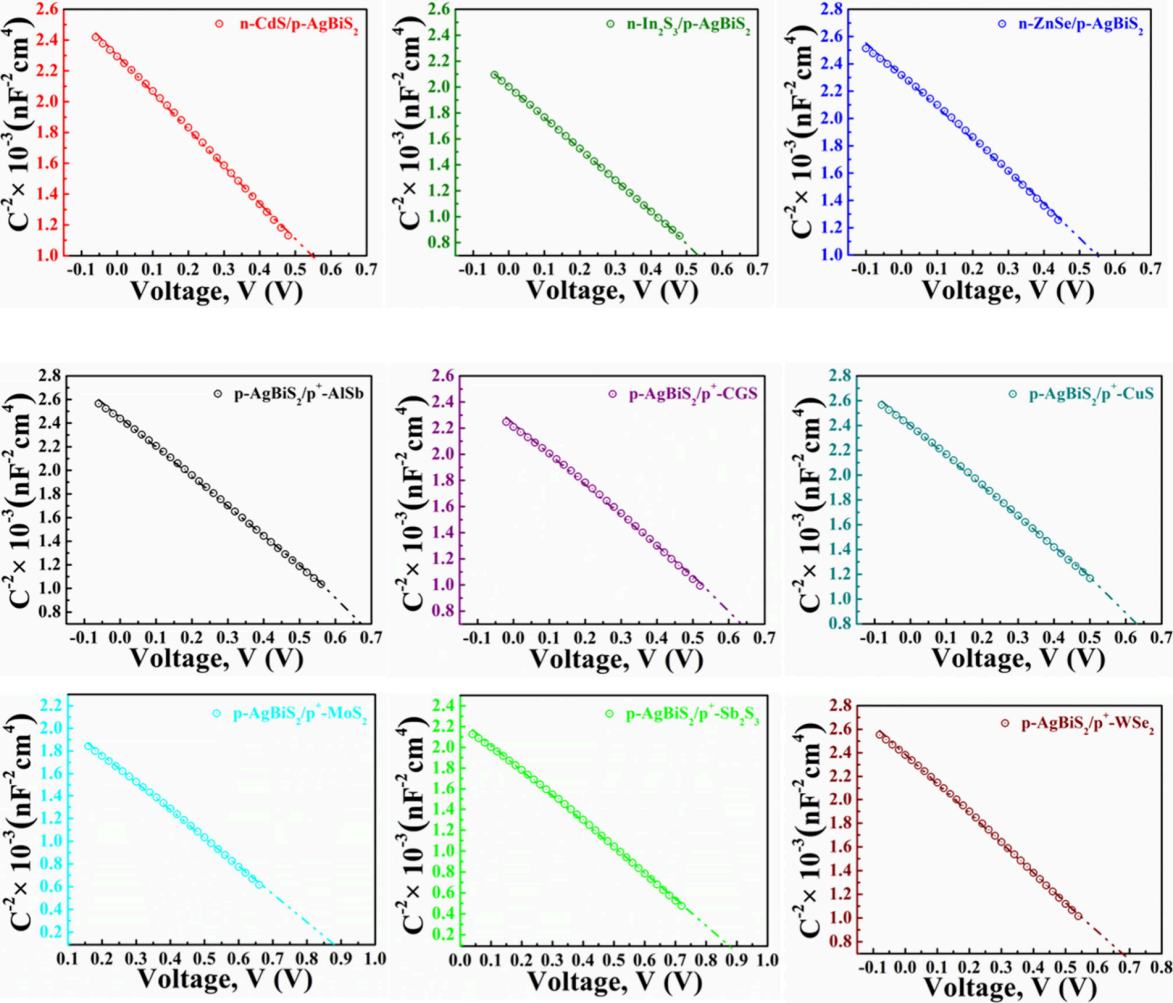
Built-in potential calculation from a *C*–*V* study for proposed AgBiS_2_ PV devices.

Figure 14 depicts the 1/*C*^2^–*V* curves for various interfaces, namely n-CdS/p-ABS, n-In_2_S_3_/p-ABS, n-ZnSe/p-ABS, p-ABS/p^+^-AlSb, p-ABS/p^+^-CGS, p-ABS/p^+^-CuS, p-ABS/p^+^-MoS_2_, p-ABS/p^+^-Sb_2_S_3_, and p-ABS/p^+^-WSe_2_. The ψ_bi_ values are obtained by fitting and projection of the linear segments present in eq 3, with the intercepts on the voltage axis providing the relevant information.

Table 3 presents the estimated and total built-in voltage values of ABS photovoltaic devices with different carrier transport layers, derived from Figure 14, facilitating a clearer comprehension. It is evident from the table that the highest overall built-in potential ψ_bi_ is achieved when employing CdS and ZnSe as a window with Sb_2_S_3_, MoS_2_, and CGS BSF layers. The generation of this elevated ψ_bi_ is attributed to the effective band alignment between the heterojunctions.^34^

**Table 3:** Built-In Potential of ABS PV Devices Using Various Carrier Transport Layers

		Built-in potential (heterojunction) (V)		
Heterostructures		With window	With BSF	Built-in potential (total) (V)
CdS/ABS	ABS/AlSb	0.55	0.67	1.22
	ABS/CGS		0.64	1.19
	ABS/CuS		0.63	1.18
	ABS/MoS_2_		0.88	1.43
	ABS/Sb_2_S_3_		0.88	1.43
	ABS/WSe_2_		0.68	1.23
In_2_S_3_/ABS	ABS/AlSb	0.54	0.67	1.21
	ABS/CGS		0.64	1.18
	ABS/CuS		0.63	1.17
	ABS/MoS_2_		0.88	1.42
	ABS/Sb_2_S_3_		0.88	1.42
	ABS/WSe_2_		0.68	1.22
ZnSe/ABS	ABS/AlSb	0.55	0.67	1.22
	ABS/CGS		0.64	1.19
	ABS/CuS		0.63	1.18
	ABS/MoS_2_		0.88	1.43
	ABS/Sb_2_S_3_		0.88	1.43
	ABS/WSe_2_		0.68	1.23

### 3.6 Overall Cell Performances of AgBiS_2_-Based Devices

**Figure 15:**
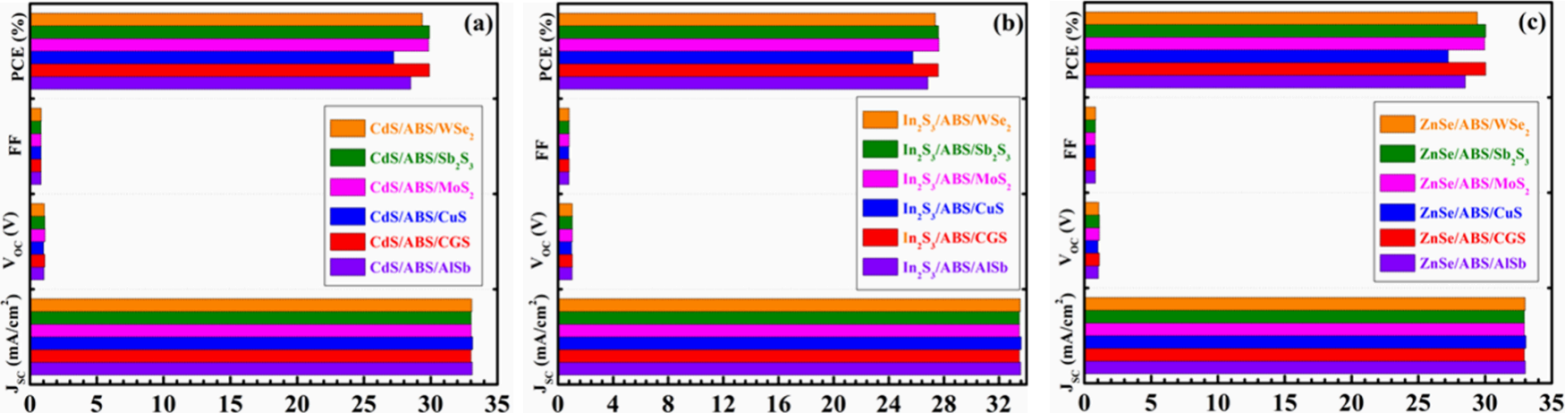
Optimum cell parameters of the modeled AgBiS_2_ based device using different BSFs and (a) CdS, (b) In_2_S_3_, and (c) ZnSe window layers.

Figure 15 illustrates the comprehensively optimized cell performances of ABS-based devices. It demonstrates that the *J*_SC_ appears to be the same in every instance. It is also evident from the figures that the highest cell performance is achieved when CdS and ZnSe are employed as window layers and MoS_2_ and Sb_2_S_3_ serve as BSF layers while maintaining ABS as the absorber layer. Notably, CGS as a BSF layer also yields PV parameters close to those obtained with the MoS_2_ and Sb_2_S_3_ BSF layers. The outstanding performance of the dual-heterostructure, composed of n-ZnSe/p-ABS/p^+^-Sb_2_S_3_, is underscored by achieving a PCE of 30.04% and a *V*_OC_ of 1.12 V. In contrast, the n-ZnSe/p-ABS/p^+^-CGS configuration displays a marginally lower PCE of 30.03% with a *V*_OC_ of 1.12 V. Additionally, the n-ZnSe/p-ABS/p^+^-MoS_2_ arrangement showcases a PCE of 29.95% and a *V*_OC_ of 1.12 V. On the other hand, the dual-heterostructure, consisting of n-CdS/p-ABS/p^+^-Sb_2_S_3_, is emphasized by achieving a PCE of 29.90% and a *V*_OC_ of 1.11 V. The n-CdS/p-ABS/p^+^-CGS configuration demonstrates a PCE of 29.90% with a *V*_OC_ of 1.11 V. The n-CdS/p-ABS/p^+^-MoS_2_ arrangement exhibited a PCE of 29.83% and a relatively high *V*_OC_ of 1.11 V. The FF reaches approximately 82% in both the ZnSe and CdS window-contained ABS solar devices utilizing any BSF. The overall efficiency of ABS devices, employing In_2_S_3_ as the window with different BSF layers, is relatively inferior compared to using ZnSe and CdS as window layers. This diminution in performance is primarily due to lower FF and *V*_OC_. The reduced FF and *V*_OC_ in In_2_S_3_ window-containing devices can be attributed to variations in diode characteristics and band alignment, respectively. Additionally, the presence of a significant fraction of nonseparated electron–hole pairs in the In_2_S_3_ region (Figure 3b) surges the dark current through recombination, thereby reducing the *V*_OC_. Specifically, the FF and *V*_OC_ values for In_2_S_3_ as the window layer with various BSFs are approximately 80% and 1.02 V, respectively.

## 4 Conclusion

The systematic exploration of a novel ABS-based solar cell, incorporating various window and BSF layers, has been revealed through the SCAPS-1D simulator. Adjustments to the physical properties of each layer, including depth, carrier concentration, and defect level, were made to enhance overall performance. Out of the three window layers, the most favorable outcomes are achieved with the ZnSe and CdS window layers. Among the six BSF layers utilizing ZnSe or CdS as the window, Sb_2_S_3_, MoS_2_, and CGS exhibited the highest PCE of 30%. These devices yield high *V*_OC_ because of their significant built-in potential, which leads to exceptional efficiency. Furthermore, with a high doping concentration of 10^19^ cm^–3^, CuS, AlSb, and WSe_2_ might deliver an equivalent efficiency with Sb_2_S_3_, MoS_2_, and CGS BSF. The In_2_S_3_ window-based ABS devices with different BSF layers have a maximum 27% efficiency. The lower FF and *V*_OC_ in In_2_S_3_ are the reasons for lowering PCE compared with CdS and ZnSe. Thus, the outcomes of this study lead to the conclusion that the ABS compound exhibits significant potential as an absorber layer in the era of solar devices. This extensive simulation of ABS photovoltaic devices using several carrier transport layers may prove useful in the future.
